# Distributions of Conception and Parturition in Dogs According to the Lunar Phase

**DOI:** 10.3390/ani15040477

**Published:** 2025-02-07

**Authors:** Jasmine Fusi, Roberta Bucci, Monica Probo, Massimo Faustini, Maria Cristina Veronesi

**Affiliations:** 1 Department of Veterinary Medicine and Animal Sciences, Università degli Studi di Milano, 26900 Lodi, Italy; jasmine.fusi@unimi.it (J.F.); massimo.faustini@unimi.it (M.F.); maria.veronesi@unimi.it (M.C.V.); 2 Department of Veterinary Medicine, University of Teramo, 64100 Teramo, Italy; rbucci@unite.it

**Keywords:** moon, lunar phases, influence, dog, mating, parturition

## Abstract

The possible effects of the Moon and its phases on parturition timing have been extensively reported in the literature in both human and animal species, sometimes finding a causal relationship and other times denying any possible influence. However, knowing parturition timing is pivotal to guarantee the best assistance to mothers and newborns, especially in polytocous species like dogs. Seventy-eight matings and related parturitions of three different canine breeds (Dobermann, Golden Retriever, and Samoyed dogs) were analyzed. Both matings and parturitions occurred more frequently in certain lunar phases, with matings mainly occurring during the Waning Moon and parturitions during the New Moon. Moreover, multiparous bitches mated more frequently during the New Moon, while primiparous ones did so during the Waxing Moon. Parturition occurred more frequently during the Waxing Moon in primiparous bitches and during the Waning Moon in multiparous ones. However, multiparous bitches bearing smaller litters experienced longer gestational lengths and more frequent parturitions during the Waxing Moon. Primiparous bitches bearing larger litters showed more frequent parturitions during the Waning Moon, with shorter gestational lengths. The results suggest that, among other factors influencing the timing of delivery, parturition in dogs can occur more frequently in certain lunar phases.

## Simple Summary

This research aimed to investigate whether mating and parturition in dogs occur more frequently in certain lunar phases. Seventy-eight matings and births in three dog breeds (Dobermann, Golden Retriever, Samoyed) were analyzed. Statistics showed that matings happened mostly during the Waning Moon, while births occurred more often during the New Moon. For dogs having their first litter, births were more common during the Waxing Moon, whereas those with previous litters gave birth more often during the Waning Moon. Additionally, the size of the litter affected gestation length and parturition timing based on the Moon phase. This study shows that matings and parturitions in dogs occur more frequently in certain lunar phases.

## 1. Introduction

Since ancient times, the Moon has compelled scientists to study its motion and associated phenomena. A lunar cycle has an average duration of 29 days, 12 h, 44 min, and 2.8 s, which is the time needed by the Moon to make a complete revolution around planet Earth [1], also defined as the time elapsing between two New Moons, or a synodic month. For research purposes, lunar phases can be grouped into four principal phases—New Moon, Waxing Moon, Full Moon, and Waning Moon—as previously reported [2].

Many natural events are influenced by lunar phases. Among all aspects, in dogs, conception and the term of pregnancy are of pivotal interest for veterinary practitioners who deal with reproduction and for breeders. The period in proximity to the Full Moon (from the end of the Waxing Moon to the beginning of the Waning Moon) was found to be statistically associated with the highest prolificity in terms of births [3]. In the human species, many studies have evaluated the possible influence of the lunar cycle on spontaneous births, with controversial results. Indeed, while some found an association between births and specific lunar phases [3,4,5], other studies reported no influence, with parturitions equally distributed among the different phases of the lunar cycle [6,7,8,9,10,11]. This variability is probably mostly due to the different populations considered, in addition to the sizes of the samples and the lengths of time considered. For this reason, in 2009, Charpentier and Causeur [12] considered parturitions occurring over 37 years and found a positive correlation with higher rates of parturition in the Full Moon phase, called the “Full Moon effect”, in women [12].

However, the exact mechanism by which the Moon acts on the onset of parturition is still unclear. Some reports focus on the gravitational force the satellite exerts when it is at the closest point to the Earth in its orbit [13]. At present, the most plausible theory is based on the effect played by the Moon on circulating melatonin concentrations, as it could have a role in triggering parturition [14,15,16,17]. Indeed, in humans, it is reported that melatonin can have a role in stimulating the onset of nocturnal uterine contractions, triggering labor [17].

An influence of the lunar phase on urinary [18] and hematic [17] melatonin concentrations in humans and finny/ichthyic species has been reported [19]. In turn, a melatonin reduction influences oxytocin levels, modulating the strengths and timing of uterine contractions, especially during nighttime [20]. In humans, oxytocin is reported to positively influence the process of fertilization: by modulating the smooth muscle contraction, it stimulates uterine and Fallopian tube motility, conveying spermatozoa through the Fallopian tubes and helping fertilization [21]. Oxytocin also has a major role in controlling parturition mechanisms in dogs [22], as reported in humans. However, unfortunately, a link between oxytocin levels and the lunar phase at parturition has not been demonstrated.

Results in bovine species have been controversial, with some studies evidencing a lunar influence on the term of pregnancy [23,24] and others reporting no effect of the Moon on the onset of parturition [25]. The possible influence of the Moon on parturition was also investigated in sheep [26], equids [27,28,29], and dromedary camels [30], with results showing the absence of a significant influence. Also, in this case, the different populations, time spans, and latitudes considered might have influenced the conclusions. Different latitudes can be subjected to different climates and weather conditions, and this may influence the onset of parturition, as previously reported for both seasonal and non-seasonal breeders [25,30].

In addition to this, the influence of the lunar phase at conception on offspring gender has been studied in literature. In canine species, one study assessed this possible influence on Labrador Retrievers and found a significantly higher male-to-female ratio when conception occurred during the Full Moon when compared to the New Moon [31]. A link between the lunar phase at conception and the male-to-female ratio was also investigated in cattle, swine, and sheep [32], equines [33], and dromedary camels [30], in all cases without finding significant effects. On the contrary, in humans, conception in the 24 h around the Full Moon was associated with increased male offspring [4,34].

A precise prediction of parturition timing is pivotal for newborn survival in dogs, as in other species, as it is essential for providing the best assistance to the mothers and puppies. From a scientific and clinical standpoint, the parturition date is predicted by fetometry, clinical and ultrasonographic monitoring of bitch and fetus parameters, and a decline in plasma progesterone concentrations [35]. Although predicting parturition should not be based on the possible influence of the lunar phase, there is still a strong belief among breeders that many births occur during the Full Moon. Therefore, the present study aimed to assess the distributions of conception and parturition in dogs according to the lunar phase.

## 2. Materials and Methods

### 2.1. Ethics

This study was conducted following the ethical guidelines provided by the animal welfare committee, and all the procedures were carried out according to the Italian legislation about animal care (DL 116, 27 January 1992) and the European Guidelines on Animal Welfare (Directive 2010/63/EU). Written informed consent was provided by each owner to record parturition data for research purposes.

### 2.2. Breeding Facilities and Animals

This study was conducted over 4 calendar years (January 2019–December 2022) in two dog breeding facilities (A and B) located in Northern Italy. Breeding facility A: located in Castellanza (VA) (latitude 45°36′38″ N, longitude 8°53′46″ E; temperature range: from −8° to +32 °C), breeds exclusively Dobermann (DOB) dogs. Breeding facility B: located in Casteggio (PV) (latitude 45°0′39.60″ N, longitude 09°7′24″ E, altitude 90 m above sea level; temperature range: from −0° to +30 °C), breeds Golden Retriever (GR) and Samoyed (SAM) dogs.

In both breeding facilities, dogs are housed outdoors, with boxes of 4 m^2^, with an external part and an internal heated one. Dogs are regularly subjected to anti-parasitic and vaccination prophylaxes and fed with commercial pet food, tailored to age and health conditions, limited to breeding females, and also based on reproductive status, and have free access to water ad libitum.

### 2.3. Reproductive Management

In both breeding facilities, as a managerial choice, natural matings with males with proven fertility are preferred. Planned on the basis of plasma progesterone concentrations, a single natural mating was performed about 48 h after the detection of values indicative of ongoing ovulation (4–12 ng/mL) [36]. After 25–28 days, pregnancy diagnosis was performed by transabdominal ultrasound examination, with measurement of the internal chorionic cavity diameters for a first birth date prediction [37]. A second ultrasound evaluation was carried out at approximately 45 days to ensure a normal course of pregnancy and the correct development and well-being of the fetuses and to measure the biparietal diameter for another estimation of the parturition date [37]. At about three weeks before the expected date of parturition, pregnant bitches were transferred to a single indoor box in specific isolated areas, equipped with a whelping box and warming devices for puppies. During the last days of pregnancy, bitches were checked daily for clinical conditions, and an ultrasound control of fetal well-being was performed. Rectal temperature was checked starting from a week before the estimated term to identify the possible decrease occurring around 24 h before the start of whelping [38]. To better predict the time of parturition, blood progesterone decreases were also monitored in the last 5 to 3 days of pregnancy [39]. With the support of dedicated cameras, all parturitions were supervised without interference, and assistance was provided only when necessary.

### 2.4. Inclusion Criteria and Data Record

For this study, only bitches that met the following inclusion criteria were enrolled: healthy bitches with normal BCS (4 or 5 on a scale of 9) [40], with a single natural mating 48 h after the detection of plasma progesterone concentrations suggestive of ovulation in progress, as previously specified; a diagnosis of pregnancy with at least 2 fetuses; a normal course and length of gestation; spontaneous eutocic birth at the physiologic end of pregnancy, with no assistance needed; and the absence of perinatal mortality.

The following data were recorded for each subject: breed, age, parity, dates of mating and parturition, pregnancy length (from the single mating), mating and parturition month and season, number of puppies per litter, sex of each newborn, and sex ratio (male/female, M:F).

### 2.5. Lunar Cycle

In this study, the main 4 lunar phases with an equal number of days were considered: Full Moon, Waxing Moon, New Moon, and Waning Moon. The lunar phases were defined based on the lunar calendar (https://www.calendario-365.it/luna/calendario-lunare.html accessed on 20 December 2018 until 10 January 2023).

### 2.6. Statistical Analysis

First, the absence of differences in clinical characteristics among the three canine breeds was assessed by a One-way ANOVA and chi-square test to allow further analysis of the total number of dogs enrolled. Subsequently, the distributions of matings and parturitions among the four lunar phases were assessed by a binomial proportion test in which the lower and upper levels at 95%CI were considered to evaluate significant differences. The overlapping of 95%CIs between groups was considered non-significant for the comparison.

After that, possible correlations of the four lunar phases at mating and at parturition with factors such as maternal age and primiparous/multiparous condition, gestational length, litter size, and male-to-female ratio (M:F) were analyzed by Multiple Factor Analysis (MFA) (R, ver 4.3.2 for Windows, package FactoMiner), which investigates the relationships between several sets of variables concurrently analyzed. Relations among variables are expressed by the coefficient of correlation (r), coefficient of determination (R^2^), and estimate (indicating the weight of a categorical variable in the MFA process) for qualitative variables. Significance was set at *p* < 0.05.

## 3. Results

During the four years of the study, 78 matings and parturitions met the inclusion criteria: 17 in 2019 (22%), 21 in 2020 (27%), 18 in 2021 (23%), and 22 in 2022 (28%). The distributions of matings and parturitions among the seasons and months, although all data were recorded, were not included in the analysis. The timing of matings is based on the breeder’s choice, so the distributions of matings and parturitions are strongly influenced by the preferences of the breeders—with decisions made to cluster births during the most favorable time of the year. The sum of matings occurring in winter/spring accounted for 49/78 cases (63%), and parturitions occurring in spring/summer accounted for 49/78 (63%).

The distributions of matings, parturitions, and the total number of puppies among the months of the year for each breed are shown in Table 1.

Data on the number and % of primiparous and multiparous bitches and the mean ± SD of maternal age, length of gestation, litter size, number of male and female puppies, and M:F are summarized in Table 2.

The chi-square test showed the absence of significant differences in the number of primiparous and multiparous bitches among the three considered canine breeds. Likewise, the ANOVA also showed the absence of significant differences in maternal age, pregnancy length, litter size, and M:F ratio. Statistical analysis was conducted on the total number of enrolled bitches.

### 3.1. Distributions of Matings and Parturitions Among Lunar Phases

The distributions of parturitions and the total number of puppies among the four lunar phases for each breed are shown in Table 3.

**Figure 1:**
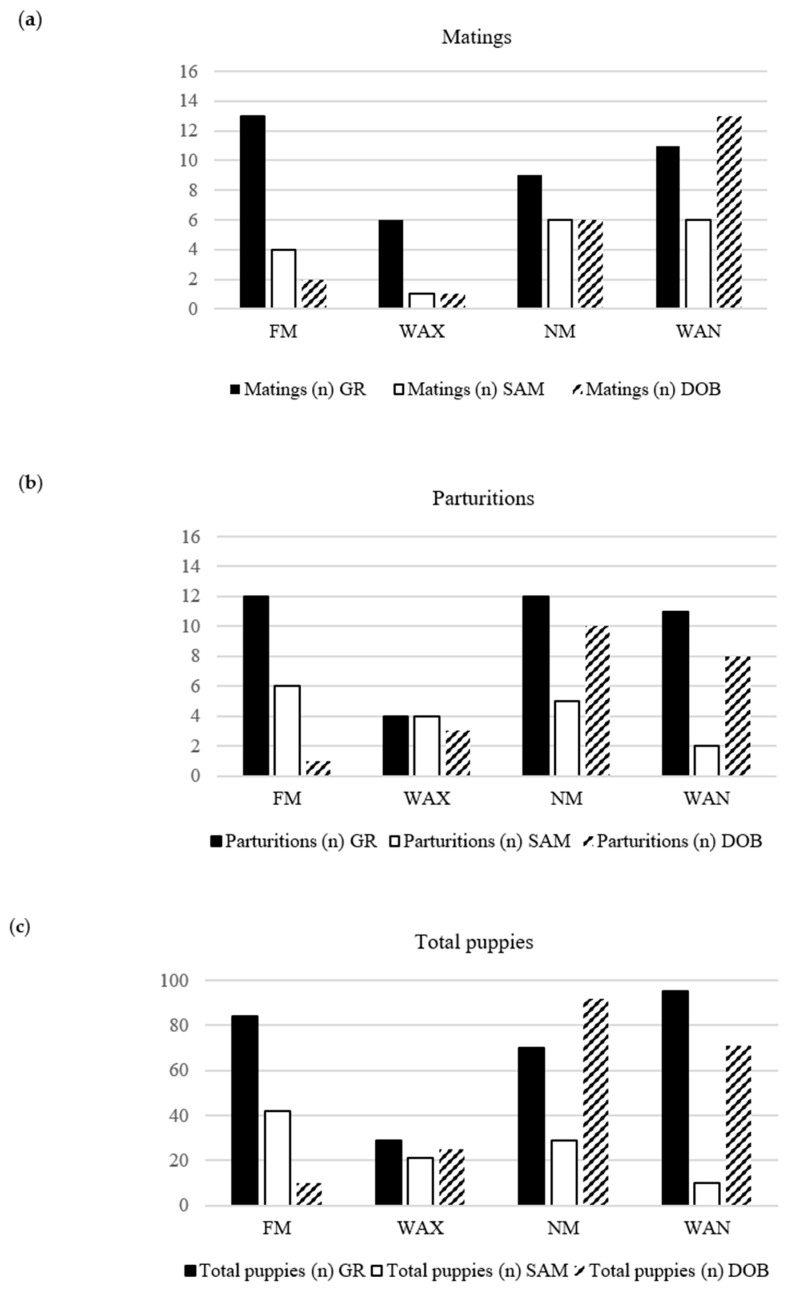
Distributions of matings (**a**), parturitions (**b**), and total number of puppies born (**c**) in the four Moon phases (Full Moon—FM; Waxing Moon—WAX; New Moon—NM; Waning Moon—WAN) according to the breed (Golden Retriever—GR; Samoyed dogs—SAM; Dobermann dogs—DOB).

The distributions of matings, parturitions, and total number of puppies born in the four Moon phases according to the breed (Golden Retriever—GR; Samoyed dogs—SAM; Dobermann dogs—DOB) are shown in Figure 1.

The distributions of matings and parturitions among the four lunar phases are shown in Table 4.

The binomial proportion test showed a significant difference in the proportion of matings among the lunar phases, with a lower proportion during the Waxing Moon and a higher proportion during the Waning Moon. According to parturition, the same analysis showed a significantly higher proportion of parturitions during the New Moon and a lower proportion during the Waxing Moon.

### 3.2. Correlations Between the Lunar Phase at Mating and Maternal Age, Parity, Gestational Length, Litter Size, and Male-to-Female Puppy Ratio

When mating was considered, the MFA evidenced that the first two dimensions explain 40.8% of the variability. The first dimension is represented by parity (correlation coefficient r = 0.89, *p* < 0.001), and parity was correlated with the lunar phase (R^2^ = 0.153, *p* < 0.01).

According to the lunar phase at mating, the MFA showed that multiparity was positively correlated with the New Moon (Estimate = 0.77, *p* < 0.01). In contrast, primiparity was positively correlated with the Waxing Moon (Estimate = 11.7, *p* < 0.001), evidencing that multiparous bitches mate more frequently during the New Moon and primiparous ones during the Waxing Moon.

In the second dimension, a positive correlation was found for gestational length (r = 0.73, *p* < 0.001) and male-to-female ratio (r = 0.24, *p* < 0.05), and a negative correlation was found for litter size (r = −0.68, *p* < 0.001).

Therefore, as the male-to-female ratio increased, the gestational length became longer, while as the litter size increased, the gestational length became shorter, and the male-to-female ratio decreased.

According to the lunar phase, negative correlations were found between litter size and the New Moon (Estimate = −0.53, *p* < 0.001) and the Waxing Moon (Estimate = −0.93, *p* < 0.01), but positive correlations were observed between the same lunar phases and the gestational length and male-to-female ratio. Positive correlations were found between litter size and the Full Moon (Estimate = 0.69, *p* < 0.05) and Waning Moon (Estimate = 0.76, *p* < 0.001), but negative correlations between the same lunar phases and the gestational length and male-to-female ratio were observed.

Mating during the New Moon or the Waxing Moon was associated with smaller litter sizes, while mating during the Full Moon or the Waning Moon was associated with larger litter sizes. Moreover, mating during the New Moon or the Waxing Moon led to longer gestations and a higher male-to-female ratio, while mating during the Full Moon or the Waning Moon led to shorter gestations and a lower male-to-female ratio. Maternal age did not show any significant correlation in the MFA for mating.

### 3.3. Correlations Between the Lunar Phase at Parturition and Maternal Age, Parity, Gestational Length, Litter Size, and Male-to-Female Puppy Ratio

When applied to parturition, the MFA showed that, similarly to what was observed for mating, the first two dimensions explain 41.3% of the variability. The first dimension was represented by parity (r = 0.85, *p* < 0.001) and gestational length (r = −0.28, *p* < 0.05), which negatively correlated with each other. These results highlighted that multiparous bitches experienced shorter gestations than primiparous ones.

According to the lunar phase, positive correlations exist between the Waxing Moon (Estimate = 0.78, *p* < 0.05) and parturition in primiparous bitches (Estimate = 11.6, *p* < 0.001) and between the Waning Moon (Estimate = 0.72, *p* < 0.01) and parturition in multiparous bitches (Estimate = 11.6, *p* < 0.001). This result suggests that parturition occurs more frequently during the Waxing Moon in primiparous bitches, while in multiparous ones, it occurs more frequently during the Waning Moon.

The second dimension was represented, in addition to parity (r = 0.36, *p* < 0.01) and gestational length (r = 0.67, *p* < 0.001), by the litter size (r = −0.68, *p* < 0.001), evidencing an influence of litter size on parity and gestational length. Therefore, as the litter size increases, the gestational length decreases in primiparous bitches. According to the lunar phase, considering the influencing effect of litter size, a positive correlation was found between primiparous bitches (Estimate = 0.28, *p* < 0.05) and parturition during the Waning Moon (Estimate = 11.3, *p* < 0.001), while multiparity (Estimate = 0.28, *p* < 0.05) was positively correlated with parturition during the Waxing Moon (Estimate = 11.0, *p* < 0.001). Therefore, multiparous bitches, when bearing smaller litters, experienced longer gestational lengths and more frequent parturitions during the Waxing Moon. Primiparous bitches bearing larger litters showed more frequent parturitions during the Waning Moon and had shorter gestational lengths. Maternal age did not show any significant correlations in the MFA for parturition.

## 4. Discussion

Parturition is a complex and multifactorial event, and its prediction requires careful pregnancy management, starting from mating. This aspect is very important for ensuring the best assistance at parturition, especially in polytocous species such as dogs, with many puppies per birth to manage. In dogs, the term of pregnancy and the event of giving birth are influenced by many factors, like body size, litter size, concomitant diseases, and, above all, the breed [41], but according to the literature, not by maternal age and parity [42,43,44]. Among exogenous factors influencing parturition in mammals, in addition to stressful conditions, to the season (in some species), and to meteorological events [23], the lunar phase, although with weak scientific evidence, is commonly believed to influence parturition, especially the Full Moon phase [3]. In the present study, all dogs experienced similar meteorological conditions, as the breeding facilities were both in Northern Italy, in the same region of Italy, with the same latitude. To date, scientific literature has provided conflicting results about the influence of lunar phases on the occurrence of parturition in humans and other animal species, with a slight majority of studies reporting an absence of statistically significant effects. It seems that not only reproduction, however, is affected by the Moon: in feral canids, for instance, it is possible to observe changes in behavioral patterns and higher vocalizations during the Waxing lunar phase [45].

Despite the scientific gap about the possible effects of lunar phases on reproduction in dogs, the belief that lunar phases influence the duration of pregnancy and therefore the timing of parturition remains strongly rooted among canine breeders. In addition to this, knowing the possible effects on parturition timing could be of pivotal importance in providing the right assistance to canine newborns. For these reasons, it deserves more interest from a scientific point of view. To date, for canine species, there is still a gap in the scientific literature, in which only the possible effect of the lunar phase on mating and the distribution of sexes of the offspring has been evaluated [31].

Data from the present study highlighted the greatest percentage of matings during the Waning Moon (39% of matings), with only 10% of the matings occurring in the Waxing Moon phase.

According to the lunar phase at mating, multiparous bitches mate more frequently during the New Moon, and primiparous ones during the Waxing Moon. Matings during the New Moon and the Waxing Moon were associated with smaller litter sizes, while matings during the Full Moon or the Waning Moon were associated with larger litter sizes. An influence of the lunar cycle on the litter size has already been reported in *Guinea pigs* [46]; however, to the authors’ knowledge, this is the first report in canine species. Moreover, mating during the New Moon or during the Waxing Moon leads to longer gestations and higher male-to-female ratios, while mating during the Full Moon or during the Waning Moon leads to shorter gestations and lower male-to-female ratios. The present data on the effect of the lunar phase on the M:F ratio is in contrast with the literature. In dogs, Alberghina and coauthors [31] described a greater M:F ratio being positively correlated with matings in the Full Moon phase. The highest probability of conceiving a male in the Full Moon phase was observed in another study, which identified the best chance of conceiving females to be 19 days after the Full Moon in humans [47]. In piglets conceived during the Waxing Moon, a significant difference in the male/female ratio from the classic 1:1 expected was seen, with a male prevalence [32]. In horses, on the other hand, no relationship between the lunar phase at mating and the sex ratio at birth was reported [33]. In human medicine, many theories about possible links between the lunar phase and sex ratio have been reported. Among these, influences of the Moon on seminal qualities, e.g., seminal volume and viscosity, on vaginal pH, and on body temperature, with a final effect on the sex ratio, have been postulated, but in none of the cases a significant influence was found [32,48]. Regarding parturitions, most of the events occurred during the New Moon (35% of total events, with 30.8% in GR, 29.4% in SAM, and 45.4% DOB), in accordance with the previous literature [31], while the Waxing Moon was the one with fewer birth events (14%). This finding corroborates the hypothesis of a lunar influence on parturition in dogs, as reported in humans and other species [3,4,5], and agrees with the highest frequency of births in the New Moon phase reported in bovines [23]. In fish, increases in melatonin receptors and melatonin levels during the New Moon, but not the Full Moon, have been reported. However, data reported in fish cannot be translated to dogs. It could be interesting to investigate whether in primiparous bitches a decrease in melatonin is associated with the Full Moon [19,49], which is not the phase with the highest number of parturitions. This would also explain why our results are in contrast with the strong belief among canine breeders that the “Full Moon effect” triggers parturition. In the present study, in contrast to what has been reported in humans [3,4,12] and bovines [24], the “Full Moon effect” was not detected. The effect of the lunar phase was mostly attributed to the moonlight rather than the gravity effects on humans [47]. In this study, assuming similar lunar conditions between the two breeding facilities, with a similar amount of moonlight, dogs tended to have a higher number of matings and parturitions during the New Moon and Waxing Moon. This could suggest a higher influence of moonlight-hour variations rather than the total number of moonlight hours, but this finding needs further research to be clarified.

The highest number of parturitions in primiparous bitches was found during the Waxing Moon, while for multiparous bitches, the highest number was found during the Waning Moon. In women, a greater influence was observed for primiparous cases during the Full Moon and for pluriparous cases in the period just after the Full Moon [3]. This finding is intriguing, even if a simple artifact cannot be ruled out [50,51,52].

Another relevant result is the shorter length of pregnancy in multiparous bitches when compared to primiparous ones, in contrast with previous reports [42,53], in which no influence of the parity status on pregnancy length was found. In addition to this, multiparous bitches with smaller litter sizes experienced longer gestations and more frequent parturitions during the Waxing Moon. On the other hand, primiparous bitches bearing bigger litters were more frequently associated with shorter gestational lengths and parturitions during the Waning Moon. The association between larger litter sizes and shorter gestational lengths—and vice versa—agrees with the previous literature [41,42,53]. However, to the authors’ knowledge, this is the first report about the different influences of the lunar phase on gestational length while also considering the parity of the bitches and the litter size.

Another finding is that all the Full Moon conceptions resulted in parturitions during the Full Moon. This result is difficult to explain, as it may only be a casual finding, given that the synodic month is composed of 29 days and 12 h. Because dog pregnancy is about 63 days [35], there is a high possibility that conception and mating will occur in the same lunar phase. On the other hand, the Full Moon could have exerted an influence in the very last phase of pregnancy. The scientific literature, however, does not report similar data in other species with different pregnancy lengths, and in dogs, data are lacking. Future research is needed to deepen this aspect.

Considering the average M:F, it is noteworthy that, in all three breeds, the number of male newborns was always greater than that of females. The M:F was 1.01 in Golden Retrievers, 1.06 in Dobermanns, and 1.04 in Samoyeds, similar to the 1.09 reported by Alberghina and coauthors [31] in Labrador Retrievers. Some studies have reported the effect of the breed on the sex ratio in different canine breeds: indeed, under the same managerial conditions, the M:F was reported to be significantly different among evaluated breeds [54,55]. However, in the present study, no significant effect of the lunar phase at parturition on the M:F was found. In dromedaries, the distribution of births showed that the male offspring has a higher probability of being born in the period from the Waning Crescent Moon to the New Moon and from the Waxing Crescent Moon to the Waxing Moon, while females are more likely to be born in the New Crescent Moon phase (in that scientific work, eight lunar phases were considered) [30].

The final consideration concerns the total number of matings and parturitions used in this study: 78 cases were studied, from three different breeds. The restrictive inclusion criteria chosen, although lowered the number of cases included, allowed a better objectivity of the data. The enrollment of bitches with only a single mating performed 48 h after progesterone plasma concentrations indicating ovulation, as previously reported [41], plays an important role. This allowed a reasonable estimation of the beginning of gestation combined with the day of the sole mating. For a similar reason, only bitches with a normal pregnancy and a normal state of health and nutrition were enrolled. Moreover, to avoid possible interference with the onset of parturition played by too-small litters, only bitches bearing litters of at least two fetuses were enrolled [56]. Furthermore, to ensure the use of data coming only from physiological situations, only bitches with spontaneous, eutocic births and without perinatal mortality were considered. The clear predominance of parturitions (over 60%) distributed in spring and summer should be interpreted only considering managerial choice.

## 5. Conclusions

In conclusion, the most relevant result of the present study is the statistical association between certain lunar phases with mating and parturition in dogs, as already reported in other species. Even if the prediction of the parturition date must be based on progesterone concentrations and ultrasonographic measurements of fetal and extra-fetal structures, being aware of an association between the lunar phase and parturition could be useful in practical breeding activity. Also, correlations among lunar phases and factors such as maternal parity, gestational length, litter size, and M:F were detected at mating. About parity, primiparous dogs with greater litter sizes showed shorter gestational lengths, while multiparous bitches with smaller litter sizes showed longer gestational lengths. Therefore, the results suggest that, among other factors influencing the timing of delivery, parturition in dogs can occur more frequently in certain lunar phases.

## Figures and Tables

**Table 1:** Descriptive data on the distributions of matings, parturitions, and the total number of puppies among the months of the year for each breed.

Month	Mating (*n*)			Parturition (*n*)			Total Puppies (*n*)		
	GR	SAM	DOB	GR	SAM	DOB	GR	SAM	DOB
**January**	3	2	2	0	2	2	0	13	14
**February**	4	2	1	1	1	2	9	9	39
**March**	5	1	6	4	1	2	25	7	0
**April**	7	0	5	4	3	1	28	13	10
**May**	7	1	0	5	1	6	37	5	60
**June**	2	1	0	6	0	5	44	0	50
**July**	1	5	0	8	1	0	56	10	0
**August**	3	2	0	0	1	0	2	7	0
**September**	4	0	3	3	5	0	15	25	0
**October**	1	0	0	3	2	0	24	13	0
**November**	0	2	3	4	0	3	33	0	15
**December**	2	1	2	1	0	1	5	0	10
**Total**	**39**	**17**	**22**	**39**	**17**	**22**	**278**	**102**	**198**

GR: Golden Retriever; SAM: Samoyed; DOB: Dobermann.

**Table 2:** Data on the number and % of primiparous and multiparous bitches and the mean ± SD of maternal age, gestational length, litter size, number of male and female puppies, and male-to-female ratio (M:F).

	Primiparous/ Multiparous *n* (%)	Maternal Age (y)	Gestational Length (d)	Litter Size	M/F (*n*)	M:F
**GR** (n = 39)	15/24 (63%)	4.5 ± 1.86	61.6 ± 1.21	7.1 ± 2.80	140/138	1.01 ± 0.74
**SAM** (n = 17)	5/12 (42%)	4.3 ± 1.45	61.9 ± 1.32	7 ± 3.50	52/50	1.04 ± 0.95
**DOB** (n = 22)	7/15 (47%)	3.8 ± 1.59	61.2 ± 1.51	9 ± 3.43	102/96	1.06 ± 0.66

GR: Golden Retriever; SAM: Samoyed; DOB: Dobermann.

**Table 3:** Descriptive data on the distributions of parturitions and the total number of puppies in the four lunar phases for each breed.

Lunar Phase	Parturitions (*n*)			Total Puppies (*n*)		
	GR	SAM	DOB	GR	SAM	DOB
**FM**	12	6	1	84	42	10
**WAX**	4	4	3	29	21	25
**NM**	12	5	10	70	29	92
**WAN**	11	2	8	95	10	71
**total**	**39**	**17**	**22**	**278**	**102**	**198**

GR: Golden Retriever; SAM: Samoyed; DOB: Dobermann; FM: Full Moon; WAX: Waxing Moon; NM: New Moon; WAN: Waning Moon.

**Table 4:** The distributions (number and %) of matings and parturitions among the four lunar phases.

	Matings			Parturitions		
	*n* (%)	95%CI for Proportion		*n* (%)	95%CI for Proportion	
		Lower	Upper		Lower	Upper
**Full Moon**	19 (24)	0.15	0.35	19 (24)	0.15	0.35
**Waxing Moon**	8 (10)	0.05 *	0.19 *	11 (14)	0.07 ^§^	0.24 ^§^
**New Moon**	21 (27)	0.18	0.38	27 (35)	0.24 ^§^	0.46 ^§^
**Waning Moon**	30 (39)	0.28 **	0.50 **	21 (27)	0.18	0.38
**Total**	78 (100)			78 (100)		

* Denotes within-row significant differences for *p* < 0.001 about matings. ** Denotes within-row significant differences for *p* < 0.05 about matings. ^§^ Denotes within-row significant differences for *p* < 0.001 about parturitions.

## Data Availability

The original contributions presented in this study are included in the article. Further inquiries can be directed to the corresponding author MP.

## References

[1] Abdul Rahman H.S. (2016). Study of the variation of the synodic month for the moon through 2000–2100. Iraqi J. Phys..

[2] Palacios C., Abecia J.A. (2011). Lunar cycle and the frequency of births in sheep. Biol. Rhythm Res..

[3] Ghiandoni G., Seclì R., Rocchi M.B., Ugolini G. (1998). Does Lunar Position Influence the Time of Delivery? A Statistical Analysis. Eur. J. Obstet. Gynecol. Reprod. Biol..

[4] Mohsin T.S. (2018). The Effect of Lunar Cycle on the Frequency of Birth in Al-Elwiya Maternity Hospital, Baghdad, 2017. Int. J. Med. Res. Health Sci..

[5] Matsumoto S., Shirahashi K. (2020). Novel perspectives on the influence of the lunar cycle on the timing of full-term human births. Chronobiol. Int..

[6] Leonard F. (1987). Childbirths and the full moon. Doings.

[7] Stern E.W., Glazer G.L., Sanduleak N. (1988). Influence of the full and new moon on onset of labor and spontaneous rupture of membranes. J. Nurse-Midwifery.

[8] Arliss J.M., Kaplan E.N., Galvin S.L. (2005). The effect of the lunar cycle on frequency of births and birth complications. Am. J. Obstet. Gynecol..

[9] Morton-Pradhan S., Bay R.C., Coonrod D.V. (2005). Birth rate and its correlation with the lunar cycle and specific atmospheric conditions. Am. J. Obstet. Gynecol..

[10] Ochiai A.M., Gonçalves F.L., Ambrizzi T., Florentino L.C., Wei C.Y., Soares A.V., De Araujo N.M., Gualda D.M. (2012). Atmospheric conditions, lunar phases, and childbirth: A multivariate analysis. Int. J. Biometeorol..

[11] Morales-Luengo F., Salamanca-Zarzuela B., Marín Urueña S., Escribano García C., Caserío Carboner S. (2020). External influences on birth deliveries: Lunar gravitational and meteorological effects. An. Pediatr..

[12] Charpentier A., Causeur D. (2009). Large-scale significance testing of the full Moon effect on deliveries. HAL Open Sci..

[13] Wake R., Misugi T., Shimada K., Yoshiyama M. (2010). The effect of the gravitation of the moon on frequency of births. Environ. Health Insights.

[14] Zimecki M. (2006). The lunar cycle: Effects on human and animal behavior and physiology. Postepy Hig. Med. Dosw..

[15] Cajochen C., Altanay-Ekici S., Münch M., Frey S., Knoblauch V., Wirz-Justice A. (2013). Evidence that the lunar cycle influences human sleep. Curr. Biol..

[16] Olcese J., Lozier S., Paradise C. (2013). Melatonin and the circadian timing of human parturition. Reprod. Sci..

[17] Koparal M., Altuntaş E.E., Yılmazer C., Altunışık E., Karataş M. (2022). Effects of the Lunar Cycle, Seasons and the Meteorological Factors on Peripheral Vertigo. Turk. Arch. Otorhinolaryngol..

[18] Law S.P. (1986). The regulation of menstrual cycle and its relationship to the moon. Acta Obstet. Gynecol. Scand..

[19] Rahman M.S., Kim B.H., Takemura A., Park C.B., Lee Y.D. (2004). Effects of moonlight exposure on plasma melatonin rhythms in the seagrass rabbitfish, Siganus canaliculatus. J. Biol. Rhythm.

[20] Russo M., Forte G., Montanino Oliva M., Laganà A.S., Unfer V. (2021). Melatonin and Myo-Inositol: Supporting Reproduction from the Oocyte to Birth. Int. J. Mol. Sci..

[21] Wildt L., Kissler S., Licht P., Becker W. (1998). Sperm transport in the human female genital tract and its modulation by oxytocin as assessed by hysterosalpingoscintigraphy, hysterotonography, electrohysterography and Doppler sonography. Hum. Reprod. Update.

[22] Veronesi M.C., Fusi J. (2022). Feline neonatology: From birth to commencement of weaning—What to know for successful management. J. Feline Med. Surg..

[23] Ammann T., Hässig M., Rüegg S., Bleul U. (2016). Effects of meteorological factors and the lunar cycle on onset of parturition in cows. Prev. Vet. Med..

[24] Yonezawa T., Uchida M., Tomioka M., Matsuki N. (2016). Lunar Cycle Influences Spontaneous Delivery in Cows. PLoS ONE.

[25] Sasaki Y., Kitai N., Uematsu M., Kitahara G., Osawa T. (2019). Daily calving frequency and preterm calving is not associated with lunar cycle but preterm calving is associated with weather conditions in Japanese Black cows. PLoS ONE.

[26] Palacios C., Abecia J.A. (2014). Does lunar cycle affect lamb production after artificial insemination in sheep?. Biol. Rhythm Res..

[27] Carluccio A., Gloria A., Veronesi M.C., De Amicis I., Noto F., Contri A. (2015). Factors affecting pregnancy length and phases of parturition in Martina Franca jennies. Theriogenology.

[28] Marinho E.N., França F.C., Santos G.S., Barbosa D.H.F., Silva Filho J.M., Palhares M.S., Lopes E.P., Viana W.S., Esquarcio L.M.G., Valle G.R. (2015). The lunar cycle influences differently the birth moment in mares according to foal sex. Rev. Bras. Reprod. Anim..

[29] Carluccio A., Veronesi M.C., Ippedico G., Contri A. (2021). Is Pregnancy Length in Martina Franca Jennies Influenced by Lunar Cycle?. Arch. Vet. Sci. Med..

[30] Iglesias Pastrana C., Navas González F.J., Delgado Bermejo J.V., Ciani E. (2023). 2023. Lunar Cycle, Climate, and Onset of Parturition in Domestic Dromedary Camels: Implications of Species-Specific Metabolic Economy and Social Ecology. Biology.

[31] Alberghina D., Gioè M., Quartuccio M., Liotta L. (2021). The influence of lunar cycle at the time of conception on sex offspring distribution in dogs. Chronobiol. Int..

[32] Abecia J.A., Arrébola F., Palacios C. (2016). Offspring sex ratio in sheep, cattle, goats and pigs: Influence of season and lunar phase at conception. Biol. Rhythm Res..

[33] Aguilar J.J., Cuervo-Arango J., Santa Juliana L. (2015). Lunar cycles at mating do not influence sex ratio at birth in horses. Chronobiol. Int..

[34] Staboulidou I., Soergel P., Vaske B., Hillemanns P. (2008). The influence of lunar cycle on frequency of birth, birth complications, neonatal outcome and the gender: A retrospective analysis. Acta Obstet. Gynecol. Scand..

[35] Beccaglia M., Alonge S., Trovo’ C., Luvoni G.C. (2016). Determination of gestational time and prediction of parturition in dogs and cats: An update. Reprod. Domest. Anim..

[36] Levy X., Fontbonne A. (2007). Determining the optimal time of mating in bitches: Particularities. Rev. Bras. Reprod. Anim..

[37] Alonge S., Beccaglia M., Melandri M., Luvoni G.C. (2016). Prediction of whelping date in large and giant canine breeds by ultrasonography foetal biometry. J. Small Anim. Pract..

[38] Veronesi M.C., Battocchio M., Marinelli L., Faustini M., Kindahl H., Cairoli F. (2002). Correlations among body temperature, plasma progesterone, cortisol and prostaglandin F2alpha of the periparturient bitch. J. Vet. Med. Ser. A.

[39] Concannon P.W., Concannon P.W., England G., Verstegen J., Linde Forsberg C. (2000). Canine Pregnancy: Predicting Parturition and Timing Events of Gestation. Recent Advances in Small Animal Reproduction.

[40] Santarossa A., Parr J.M., Verbrugghe A. (2017). The importance of assessing body composition of dogs and cats and methods available for use in clinical practice. J. Am. Vet. Med. Assoc..

[41] Okkens A.C., Teunissen J.M., Van Osch W., Van Den Brom W.E., Dieleman S.J., Kooistra H.S. (2001). Influence of litter size and breed on the duration of gestation in dogs. J. Reprod. Fertil. Suppl..

[42] Eilts B.E., Davidson A.P., Hosgood G., Paccamonti D.L., Baker D.G. (2005). Factors affecting gestation duration in the bitch. Theriogenology.

[43] Gavrilovic B.B., Andersson K., Linde Forsberg C. (2008). Reproductive patterns in the domestic dog--a retrospective study of the Drever breed. Theriogenology.

[44] Mir F., Billault C., Fontaine E., Sendra J., Fontbonne A. (2011). Estimated pregnancy length from ovulation to parturition in the bitch and its influencing factors: A retrospective study in 162 pregnancies. Reprod. Domest. Anim..

[45] Ferreira L.S., Rocha L.H.S., Duarte D., Neto E., Baumgarten J.E., Rodrigues F.H.G., Sousa-Lima R.S. (2020). Temporal and spatial patterns of the long-range calls of maned wolves (*Chrysocyon brachyurus*). Mastozool. Neotrop..

[46] Perea M.F., Fernández E.A., Garzón J.P., Rosales C.A., Hernández-Fonseca H., Perdomo D.A., Perea F.P. (2024). The moon cycle influences reproductive and productive traits in guinea pigs (*Cavia porcellus*) from a tropical Andean area. Chronobiol. Int..

[47] Onken D., Marty E., Palomares R., Xie R., Zhang L.R., Arnold J., Gutierrez J.B. (2017). The lunar cycle’s influence on sex determination at conception in humans. arXiv.

[48] Moretti E., Tallis V., Trovarelli S., Gnech M., Capitani S., Ponchietti R., Collodel G. (2008). Do lunar phases influence semen parameters?. J. Androl. Sci..

[49] Ikegami T., Motohashi E., Doi H., Hattori A., Ando H. (2009). Synchronized diurnal and circadian expressions of four subtypes of melatonin receptor genes in the diencephalon of a puffer fish with lunar-related spawning cycles. Neurosci. Lett..

[50] Miyaoka E. (1989). Application of mixed Poisson-process models to some Canadian data. Can. J. Stat..

[51] Kelly I.W., Martens R. (1994). Geophysical variables and behavior: LXXVIII. Lunar phase and birthrate: An update. Psychol. Rep..

[52] Maschio E., Soldani R., Cagnacci A., Falqui A., Melis G.B., Melis G.B. (1994). Il ritmo circadiano del parto è modulato dalle stagioni, differenze fra primipare e pluripare. Endoscopia Chirurgica in Ginecologia. Domus de Maria.

[53] Okkens A.C., Hekerman T.W., de Vogel J.W., van Haaften B. (1993). Influence of litter size and breed on variation in length of gestation in the dog. Vet. Q..

[54] Tedor J.B., Reif J.S. (1978). Natal patterns among registered dogs in the United States. J. Am. Vet. Med. Assoc..

[55] Gharagozlou F., Youssefi R., Akbarinejad V. (2016). Effects of diets supplemented by fish oil on sex ratio of pups in bitch. Vet. Res. Forum.

[56] Münnich A., Küchenmeister U. (2009). Dystocia in numbers—Evidence-based parameters for intervention in the dog: Causes for dystocia and treatment recommendations. Reprod. Domest. Anim..

